# Dissonant views - GPs’ and parents’ perspectives on antibiotic prescribing for young children with respiratory tract infections

**DOI:** 10.1186/s12875-019-0936-5

**Published:** 2019-03-28

**Authors:** Ruby Biezen, Danilla Grando, Danielle Mazza, Bianca Brijnath

**Affiliations:** 1 Department of General Practice, Monash University, Building 1, 270 Ferntree Gully Road, Notting Hill, VIC 3168 Australia; 2 School of Science, RMIT University, Building 223, Level 1, Bundoora Campus, Plenty Road, Bundoora, VIC 3083 Australia; 3 National Ageing Research Institute LTD, 34-54 Poplar Road, Parkville, VIC 3052 Australia

**Keywords:** Antibiotic prescribing, Antimicrobial resistance, Respiratory tract infection, Children, General practitioners, Parents, Primary care

## Abstract

**Background:**

Antibiotics are not recommended for treating uncomplicated respiratory tract infections (RTIs), despite this, antibiotic prescribing for this is widespread. General practitioners (GPs) report parental pressure and fear of losing patients if they do not prescribe antibiotics, however, parental views on antibiotics for RTIs are unclear. Therefore, this study examined GPs’ and parents’ perceptions regarding antibiotic prescribing for RTIs in young children.

**Methods:**

We conducted semi-structured interviews with 20 GPs, and a survey and focus groups with 50 parents and carers of children under the age of five between June 2014 and July 2015 in Melbourne, Australia. Qualitative data were thematically analysed using NVivo and quantitative data were analysed using SPSS.

**Results:**

GPs believed that parents expect antibiotics for RTIs and were more likely to prescribe them if parents were insistent. They believed parents would go elsewhere if they did not prescribe antibiotics. GPs suggested that there would be less conflict if parents were better educated on appropriate antibiotics use.

In contrast, parents demonstrated good knowledge of RTIs and appropriate antibiotic use. Their main expectation from GPs was to obtain a diagnosis, discuss management, and receive reassurance that the illness was not serious. Parental satisfaction with GPs was not dependent on receiving antibiotics (r = 0.658, *p* < 0.001), and they would not seek another GP if antibiotics were not prescribed (r = 0.655, *p* < 0.001).

**Conclusion:**

GPs and parents have dissonant views on antibiotic prescribing for RTI in young children. GPs perceived parents wanting a diagnosis and reassurance that their child is not severely ill as pressure to prescribe antibiotic. To overcome these barriers, targeted training for both GPs and parents to improve communication and reassurance that satisfaction is not related to receiving antibiotics may reduce unnecessary antibiotic prescribing for RTI in young children.

**Electronic supplementary material:**

The online version of this article (10.1186/s12875-019-0936-5) contains supplementary material, which is available to authorized users.

## Background

The overuse of antibiotics has led to antimicrobial resistance globally, posing immediate and long-term threats [1, 2]. It is estimated by 2050, most antibiotics will be useless against common bacterial infections, leading to an annual loss of 10 million lives [2]. Antibiotics are ineffective in the combat of viral infections, and do not appear to provide clinical benefits in the management of uncomplicated respiratory tract infections (RTIs). They have also been shown to cause adverse effects such as diarrhoea and rash [3–6].

Recent research on antibiotic prescribing for RTIs in Australian general practice found rates 4–9 times higher than those recommended by national guidelines [7]. Similar rates were found from antibiotic prescribing for children in Australia [8], while studies from the UK have also shown higher than recommended rates of antibiotic prescribing in the community [9, 10]. Reasons identified for inappropriate prescribing for RTIs from physicians included parental misconception regarding indications for antibiotic use, parents’ expectation of antibiotics, perceived parental pressure, and diagnostic uncertainty of RTIs especially in young children [11–18]. However, studies from parents’ perspectives have found that parents mostly wanted a diagnosis and did not necessarily want antibiotics [19, 20]. There seems to be a discord between physicians’ perception of what parents expect and what parents really want when it comes to prescribing and receiving antibiotics.

Currently, only a limited number of studies have explored the contrasting views between general practitioners (GPs) and parents of young children with RTIs, and fewer studies have been identified in the literature that have explore contrasting views using a mixed methods approach. Therefore, the aim of this study was to compare GPs and parents’ views on antibiotics for RTIs in young children, exploring barriers and contrasting views by using quantitative and qualitative methods.

## Methods

A mixed methods cross-sectional design was applied to understand GPs and parents’ views on their knowledge and attitudes towards prescribing antibiotics for RTIs in young children. The qualitative component comprised semi-structured interviews with 20 GPs and five focus group discussions with 50 parents and carers of young children (see Additional file 1). In addition, a short questionnaire (see Additional file 2) was provided for parents who participated in the focus groups. For validity, interview questions were piloted with two GPs, and focus group questions were piloted with two parents of children < 5. Data from the pilots were not included in the final analysis.

Detailed description of the recruitment and sampling framework applied have been described elsewhere [21]. In summary, interviews were conducted with 20 GPs and five focus groups with 50 parents of young children to explore their views, knowledge and attitudes towards management of respiratory tract infections, including prevention strategies such as influenza vaccination and hand hygiene in children < 5 years of age. (See Additional file 1).

GPs were recruited via a diversified sampling strategy, with contact details of GPs generated from an existing general practice database at Monash University, Victoria, Australia. Recruitment was limited to one GP per practice site across metropolitan Melbourne, Australia. Purposive sampling via advertisements was used to target parents and carers from the south east and east of Melbourne, Australia. In total, the researcher (RB) approached a total of five playgroups (two accepted) and three mothers’ groups (all three accepted), to discuss the study in detail. All participants signed the consent form (up to an hour interview or focus group) before participating in the study. Interviews were conducted at the GPs’ practice and focus groups were conducted at the playgroups or mothers’ group by RB, an experienced qualitative researcher. GPs and parents were provided with a AUD$120 and a AUD$40 gift voucher respectively upon completion.

Interviews and focus group discussions were digitally recorded and transcribed verbatim. Data were analysed using a thematic approach [22]. Initially, two researchers (RB and BB) read three transcripts independently to generate initial codes and themes, which were then compared and refined until consensus was reached. A further three transcripts were coded using the schemata and this process was repeated, three transcripts at a time, to incorporate emerging themes, until all transcripts were coded. Data were managed using NVivo10.

Parents were asked to complete the anonymous questionnaire (See Additional file 2) before taking part in the focus group discussions. Data were analysed using SPSS version 20 Statistics package. Logistic regression was used to analyse predictors of knowledge questions, and the strength and direction of the relationship between two variables were analysed using Spearman’s Rank Order Correlation.

Study approval was obtained from Monash University Human Research Ethics Committee (CF14/1384-2014000648).

## Results

A total of 20 GPs and 50 parents and carers participated in the study between June 2014 and July 2015. Characteristics of GPs and parents and carers are showed in Tables 1 and 2 respectively.

**Table 1:** Characteristics of GPs

	GPs (*n* = 20)
Gender	6 F, 14 M
Age group	31–40 (30%)
	41–50 (20%)
	51–60 (45%)
	61–70 (5%)
Years of experience	18 (4–37 years)

*GPs* General Practitioner, *F* Female, *M* Male

**Table 2:** Characteristics of parents and carers

Parents and Carers	*N* = 50
Gender	
Women	47 (94%)
Men	3 (6%)
Age: (year)	
21–30	7 (14%)
31–40	31 (62%)
41–50	9 (18%)
> 50 (grandparents)	3 (6%)
Income (per week)	
< $900	6 (12%)
$900–$1500	14 (28%)
> $1500	24 (48%)
No data	6 (12%)
Highest qualifications	
High school/trade certificate	14 (28%)
Undergraduate	22 (44%)
Post graduate/PhD	14 (28%)

### Results from parents’ questionnaire

In terms of what parents expected from their GP visit during a RTI consultation, a third (66%, *n* = 33) responded that they wanted a diagnosis, management advice and/or reassurance that they were doing the right thing; while 22% (*n* = 11) wanted medication (over-the-counter medication, not antibiotics), only 8% (*n* = 4) sought and expected antibiotics. From the knowledge questions, the majority (84%, *n* = 42) correctly answered that most cough, cold and flu illness were caused by viruses, and that antibiotics were needed for bacterial and not viral infections (72%, *n* = 36).

Neither parental age, profession, qualification nor income were found to associate with parental knowledge.

When we asked parents about what they expected from their GP, 86% (*n* = 43) strongly disagree/disagree that they wanted antibiotics for their child’s RTIs symptoms (Table 3). Parents’ satisfaction of GP visits was not dependent on prescribing of antibiotics for their child’s cough, cold and flu symptoms (*r =* 0.658, *n* = 50, *p* < 0.001). Similarly, most parents (88%, *n* = 44) strongly disagree/disagree that they would go to another doctor if antibiotics were not prescribed for their child’s RTI symptoms (r = 0.655, n = 50, *p* < 0.001) (Table 3).

**Table 3:** Parents’ views on common colds, antibiotics and GP visits

Statements	Disagree or Strongly disagree (n, %)		Neither agree or disagree (n, %)		Agree or Strongly agree (n, %)		No Response (n, %)	
I will always want antibiotics for my child’s cough, cold or flu symptoms	43	86%	4	8%	1	2%	2	4%
My child will be sick for a longer period if he/she does not receive an antibiotic for cough, cold or flu symptoms	40	80%	4	8%	5	10%	1	2%
I generally know if my child needs an antibiotic before seeing the doctor for cough, cold or flu symptoms	17	34%	9	18%	23	46%	1	2%
I will go to another doctor if my doctor does not prescribe antibiotics for my child for cough, cold or flu symptoms	44	88%	4	8%	1	2%	1	2%
I usually go to the doctors if my child has been unwell with cough, cold or flu symptoms for longer than 3 days	17	34%	7	14%	25	50%	1	2%
I am more satisfied with the doctor visit if I am prescribed antibiotics for my child with cough, cold or flu symptoms	36	72%	7	14%	6	12%	1	2%
I am always guided by what my doctor recommends for my child with cough, cold or flu symptoms	8	16%	6	12%	35	70%	2	4%
I always have to initiate the discussion of antibiotics before my doctor would be willing to prescribe antibiotics for my sick child with cough, cold or flu symptoms	32	64%	20	20%	6	12%	2	4%
I usually know what I want out of my doctor’s appointment before I go	13	26%	17	34%	19	38%	1	2%
I will take my child to see a doctor if he/she has a high temperature (over 40 °C)	4	8%	3	6%	41	82%	2	4%
I will take my child to see a doctor if he/she has a temperature (37 °C - 40 °C)	18	36%	12	24%	29	38%	1	2%

From the survey results, parents showed good knowledge and understanding of what antibiotic was used for. They also expressed they wanted a diagnosis of the child’s illness in an RTI consultation and that they did not necessarily want antibiotics and would not go elsewhere if antibiotic was not prescribed. However, to further understand the views of GPs and parents and carers on their knowledge and practice on antibiotic prescribing for the common cold, we compared and contrasted findings from the interviews and focus groups.

### Results from GPs and parents’ interviews and focus groups

#### Theme 1 - “…they don’t understand the difference between viral and bacterial…”

GPs’ experience with parents coming in with a sick child for an RTI consultation varied according to the symptoms of the child, the anxiety level of the parent, and GPs’ perception of parents’ expectation of the visit. Amongst these variables, GPs thought the lack of knowledge in some parents forced some GPs to prescribe antibiotic unnecessarily.

*“… they probably think that it will fix the child, that’s why they ask for antibiotics…” GP12*

*“There’s poor understanding and they’re not scientifically equipped to really understand what it means.” GP6*

Due to the perceived lacked of knowledge, GPs thought educating these groups of parents would greatly reduce the pressure and the need to prescribe antibiotics.

*“… if you educate them early and you have much less of a fight later on down the track.” GP13*

However, parent participants were knowledgeable on what antibiotics were used for and possible iatrogenic effects. Some insisted on no antibiotics even upon recommendations from their GP.

*“I know antibiotics are not good. It kills the good bacteria as well as the bad bacteria…” FG2*

While most parents showed good general knowledge of common colds and antibiotics, a small number did confuse bacteria with viruses, with some stating that viruses were not associated with infections. In addition, some parents misinterpreted GPs’ advice and recommendations, leading to miscommunication and further confusion for parents regarding antibiotics.

*“… we thought it was an infection and they’re like no, it’s a virus, but have antibiotics. We’re like - he gave us the script, we just never got it filled… Because we were like well, if it’s a virus what’s the point of the antibiotics…” FG1*

#### Theme 2 – “… if they’re angry they might not come back…”

GPs’ concern that patients would go elsewhere if antibiotics were not prescribed was a common barrier to appropriate antibiotic prescribing. GPs said they preferred to prescribe antibiotics to please the patients rather than seeing them go elsewhere:

*“… if they are absolutely insistent and I know they are pretty much going to walk out of the door and request for another doctor, I’d give them a script…” GP13*

In additions to the fear of losing patients, GPs commented that parental pressure and expectations of wanting antibiotics for their sick child influenced some GPs to prescribe antibiotics unnecessarily.

*“… some parents do have an expectation having antibiotics when they come along…” GP5*

Ultimately, it was about keeping parents happy:

*“…a lot of the GPs are trying to please the patients and I think they worry about the child, so you know, the person comes along and think they should give the child antibiotics…” GP5*

In contrast, parents expressed that they did not expect antibiotics for a child with a common cold, instead preferring a diagnosis and reassurance that their child would recover.

*“I’ve seen a doctor before and she’s prescribed, for my daughter who was seven months at the time, to have antibiotics. She didn’t need antibiotics. I knew what she had was viral, I just wanted… the reassurance.” FG5*

*“I think the only reason that we would go to doctors is simply to make sure there’s no infection in the chest or ears, throat, anything like that…” FG1*

Hence while GPs were worried about pleasing and satisfying parents, most parents only needed reassurance that their child was not seriously ill.

#### Theme 3 - “… there are two ways to do this [antibiotic prescribing], there is the quick way, and the right way…”

One of the biggest barriers to appropriate antibiotic prescribing was GPs’ misconception that it took longer to explain why antibiotics might not be appropriate, than to issue a script. GPs thought while the conversation with parents was important, it was easier to prescribe if they were running behind and leave the conversation for ‘next time’.

*“… it takes a very long time. Much easier to write a script.” GP1*

*“…if I’m 40 mins behind, and I want to catch up, and they (parents) are naggy, there might be something that I would prescribe this time around, and readdress that for the next time…” GP13*

In addition, diagnostic uncertainty, especially in a young child, as well as a fear of possible litigation, forced GPs to ‘play it safe’ when it came to prescribing antibiotics to a young child with an RTI.

*“…sometimes you’re influenced by... it’s the third time this week, and you feel they (parents) want you to do something and you feel that you should do something and you think well, you know, it is the third time this week, maybe it isn’t viral, maybe it’s something serious...” GP7*

Therefore, GPs faced many barriers leading to the decision to prescribe or not to prescribe antibiotics to a young child with an RTI. However, parents reported they generally placed their trust in their GPs and hence happy for the discussion to take place within the consultation.

*“I’m really guided by my GP... I really go by what they say…” FG4*

*“I don’t understand any of the science behind it and I would go ‘Okay, if that’s what you recommend, you’re the professional’. I wouldn’t even question it.” FG5*

#### Theme 4 –“…everyone knows, right? Whether you practice it or not, that’s the main thing…”

Analysis of the qualitative data highlighted some enablers to reduce inappropriate antibiotic prescribing: GPs strongly thought that parents needed to be educated to increase their knowledge of common colds and usefulness of antibiotics, therefore minimising the need for parents to demand antibiotics and GPs having to deal with difficult situations.

GPs also recognised that educating GPs on how to handle difficult parents and situations would improve the communication between both parties.

*“[Educate GPs on] how to say no, how not to offend somebody, how to educate them at the same time, in a short space of time, so I do think there is a need for that…” GP1*

Most GPs reported using delayed prescribing as a common method to address inappropriate antibiotic prescribing.

“*I would say to them, “I firmly believe that it is not bacterial and that your child doesn’t need antibiotics, and by giving it, you’re not helping [him or her], you’re actually reducing their immunity and creating resistance”. However, they are the parents and guardians of the child, I do not want to not give them the script, I would give it to them and say, “Please use it vigilantly”. I would explain to them what the symptoms are… I’m not going to totally not give the script, and go against them head on…” GP12*

Ultimately, GPs believed that constant reminders on appropriate prescribing and the harm of over prescribing would sustain good prescribing behaviour.

Parents, on the other hand, said they would welcome information on what to look for in terms of common cold symptoms, and when antibiotics should be prescribed. They expressed this approach would provide parents with the knowledge and confidence to refuse antibiotics even if antibiotics were to be prescribed.

*“Just talk about the common cold and things that these mothers might come across with their children and then suggest the possible antibiotics they can possibly choose from and talk about the illnesses themselves first before the cures…” FG3*

*“I think education, like first time Mums is key, helping them to understand… If they understand their child’s experience… [including] symptoms and a range of degrees of unwellness, it would be good…” FG2*

In the end, both parents and GPs agreed that better communication would be the best strategy to increase understanding between parents and GPs to best reduce antibiotic prescriptions in the community. Parents wanted GPs to discuss management options instead of just prescribing antibiotics.

## Discussion

This is the first study using a mixed methods approach to compare and contrast views from GPs and parents in regards to antibiotic prescribing for RTIs in young children. GPs identified many barriers to reducing inappropriate prescribing including parental lack of knowledge regarding the common cold and antibiotics, parental pressure and expectation, diagnostic uncertainty of RTIs in young children, and time constraints in an RTI consultation. GPs felt this forced them to prescribe antibiotics at times deemed inappropriate. In contrast, our parent cohort showed good knowledge of common colds and what antibiotics were used for, and they mostly just wanted a diagnosis and reassurance that their child was not seriously ill rather than wanting or expecting antibiotics. From both the parent qualitative and quantitative findings, it was clear that parents do not always want antibiotics; satisfaction with their GP visit was not dependent on receiving antibiotics; and they would not go elsewhere if they were not prescribed an antibiotic for their child with an RTI.

Parental knowledge has been identified as an important influence on when and how to use antibiotic for RTIs in young children [23, 24]. GPs in our study expressed that parents did not understand antibiotic use, often linking antibiotics as a cure for the common cold. In contrast, our quantitative analysis showed that our parents scored high on the knowledge questions on what caused the common cold, what antibiotics were used for, and were knowledgeable in this area. This study highlighted contradicting insights regarding knowledge of common colds and antibiotics among GPs and parents.

Previous studies have shown that GPs believed parents have an expectation of antibiotics before coming in for an RTI consultation [16, 25–31]. This belief often led to inappropriate prescribing and/or ‘just in case’ prescribing, even when GPs knew antibiotics were not warranted. Mangione-Smith et al., suggested that physicians interpret parental questioning of a treatment plan as wanting antibiotics for their sick child [16], therefore the perception of patients’ desire for antibiotics was strongly associated with antibiotic prescribing [27, 30]. This is consistent with our findings that GPs interpreted parents’ attitude as wanting an antibiotic, while parents merely wanted a diagnosis and confirmation that their child was not seriously ill.

Our study also demonstrated that some GPs were concerned if antibiotics were not prescribed, parents might seek them elsewhere. However, both the quantitative and qualitative data confirmed that parents’ satisfaction was not dependent on receiving antibiotics and that they would not go to another GP if antibiotics were not prescribed. Studies have shown that clinicians’ perceptions did not always match patient views, in that patients were generally satisfied with care [32], and sought medical evaluation and decisions, while clinicians wanted satisfied parents and short consultations [26]. It is therefore important to emphasise communication between GPs and parents is paramount if inappropriate prescribing is to be reduced. A possible barrier mentioned by GP participants to effective communication was the lack of consultation time needed to educate parents on appropriate antibiotic treatment for RTIs. GPs commented if they were running behind, or if it was late in the afternoon or on the weekend, they were more likely to prescribe and less likely to counsel patients. However, some studies did not support the view that it took longer to counsel and educate patients than to prescribe an antibiotic [33, 34], underlying the importance of taking time to communicate and educate parents, and reassuring GPs that parents did not necessary want or expect antibiotics.

One of the methods GPs used to combat barriers such as parental pressure, expectations and time constraint was delayed prescribing. The process of suppling a script to parents and telling them to only fill it if the symptoms did not get better after a couple of days, have certainly been shown to reduce antibiotics use [35–37], and this was also mentioned as part of a strategy to overcome parental pressure and expectations in our study. However, our study also showed that it confused our parents as to why a script was provided while GPs suspected the RTI was viral in origin. Even though this has been a preferred method for many primary care physicians, studies have emerged that delayed prescribing may provide opportunities for patients to store antibiotics and/or unfilled scripts at home for later use [24, 38], leading to inappropriate use, and possible antimicrobial resistance (AMR). A recent study suggested that delayed prescribing may also give GPs ‘permission’ to prescribe, which may increase overall antibiotic prescribing [39]. While delayed prescribing might be used as a ‘safety net’ for GPs to overcome patient pressure and expectations, and increase patients’ ‘shared decision making’, this approach will pass the responsibility back to the parents. Given that GPs thought parents were not ‘knowledgeable’, expecting parents to do the ‘right’ thing with an antibiotic script would seem unreasonable. It should then be argued, that educating parents in the first place, and further emphasising the importance of communication between GPs and parents, and to take the time to explain to parents as to why antibiotic was not necessary for RTIs, would be the preferred option to delayed prescribing.

Our study demonstrated that parents mainly placed their trusts in their GPs, whether they agreed with the GPs’ treatment decision and would accept the script instead of questioning the GP. A recent study found that the majority of their parents would accept their clinicians’ management decisions regardless of whether antibiotic was prescribed [40]. If GPs can accept that parents do not expect or want antibiotics, and also parents will trust them with the treatment management for their sick child, they should be reassured that parents are satisfied and will not seek antibiotics elsewhere.

Communication between GPs and parents is therefore a vital component to reducing antibiotic prescribing in general practice. Having a conversation to discuss why antibiotic was or was not needed and involving patients in the decision-making process of treatment management, may further reduce inappropriate antibiotic prescribing [41–43].

The high income and level of qualifications of our parent participants as well as the lack of male parent participants were limitations of our study. This group of parents were not a true representation of socio-economic status of the average Australian family, therefore this may have biased and overrated the knowledge component of this study. These parent participants represented a convenient/opportunistic data set rather than a true sample size needed for a significant representation. In addition, providing incentives to participants may have led to a possible source of bias, although these incentives are aligned with similar work with estimated earnings and average Australian wage [44, 45]. Finally, parents’ views may have been vastly different if they were recruited at a general practice with a sick child as opposed to a calmer and happier playgroup/mothers’ group environment.

One of the strengths in our study was the ability to obtain views from both parents and GPs and comparing and contrasting their knowledge and practice using a mixed methods approach. Using this approach, we were able to identify areas of discord between these two groups and guide the development of interventions that could bridge the communication gap between GPs and parents.

This study has identified a number of areas where interventions can be targeted to better manage RTIs in this cohort. In order to assist GPs in appropriate antibiotic prescribing decisions and increase parental knowledge and better understanding of the use of antibiotics, the following interventions should be considered: 1) implement public health campaigns to raise awareness and increase knowledge of antibiotic use for parents; 2) provide support for GPs to improve better communication with parents, hence reaching the understanding that parents want reassurance rather than antibiotics; and 3) reassure GPs that prescribing antibiotics is not necessarily associated with satisfaction of the visit, and parents will not necessarily go elsewhere if antibiotic was not prescribed. Ultimately, parents mainly place their trust in GPs and appropriate antibiotic prescribing can be accomplish with better communications between GPs and parents to achieve better understanding of what parents really want, hence over all, reducing inappropriate antibiotic prescribing.

## Conclusions

Our study demonstrated dissonant views exist between GPs and parents around antibiotic prescribing and use for RTIs in young children, contributing to unnecessary prescribing and use of antibiotic in this cohort. Both qualitative and quantitative studies demonstrated that parents showed good knowledge of common colds and antibiotic use, did not expect or wanted antibiotics, and will not go elsewhere if antibiotics were not prescribed. However, dissonance emerged where GPs perceived parents wanting antibiotics, and concern that parents would go elsewhere if antibiotic was not prescribed; hence linking satisfaction to antibiotic prescribing. GPs should be reassured that parents do not necessarily want antibiotics, and they generally place their trust in their clinical judgement. Training on how to handle anxious parents may also be beneficial to GPs to alleviate their concerns. Better communication between GPs and parents is needed to minimise this discord in order to achieve appropriate prescribing of antibiotics and increase better health outcome for RTIs in young children.

## Additional files

Additional file 1: Interview and focus group schedules. Interview questions for GPs and focus group questions for parents and carers. (DOCX 14 kb)

Additional file 2: Parents questionnaire. Questionnaire for parents and carers. (DOCX 21 kb)

## Abbreviations

FG: Focus groups
GP: General practitioner
MCHN: Maternal and child health nurse
PCP: Primary care providers
PN: Practice nurse
RTIs: Respiratory tract infections
