# Supplementary material for: Dissonant views - GPs’ and parents’ perspectives on antibiotic prescribing for young children with respiratory tract infections
Source: BMC Fam Pract. 2019 Mar 28;20:46. doi: 10.1186/s12875-019-0936-5 (PMC6437946; doi:10.1186/s12875-019-0936-5)
Supplement: Supplementary file 1 — Interview and focus group schedules. Interview questions for GPs and focus group questions for parents and carers. (DOCX 14 kb) [file 12875_2019_936_MOESM1_ESM.docx]

## Interview and focus group schedules

**GPs’ interview schedule:**

1. How do you manage children’s cold symptoms?
2. What do you recommend to the parents/carers?
3. Can you tell me the process of prescribing antibiotics for respiratory tract infections in this age group?

Prompts:

- Recommend over the counter medications, type of over the counter medications recommended
- Under what circumstances is antibiotics prescribed?
- Referring patients to specialists

1. Are you guided by parents (re. antibiotics)?

Prompts:

- In terms of counselling

1. Do you think it is worthwhile having an education program for parents on antibiotics? How would you like to see it implemented?
2. Do you see a need for an education program for GPs and practices on antibiotics? How would you like to see it implemented?

**Parents’ focus group schedule:**

1. What do you do when a child has a cold?
2. How would you manage a child’s cold symptoms?

Prompts:

- Over the counter medications
- Antibiotics

1. What do GPs recommend? Would you consider asking for medications?

Prompts:

- Over the counter medications
- Antibiotics

1. Do you think it is worthwhile having an education program for parents on antibiotics? Do you think it is possible?
