# Supplementary material for: Dissonant views - GPs’ and parents’ perspectives on antibiotic prescribing for young children with respiratory tract infections
Source: BMC Fam Pract. 2019 Mar 28;20:46. doi: 10.1186/s12875-019-0936-5 (PMC6437946; doi:10.1186/s12875-019-0936-5)
Supplement: Supplementary file 2 — Parents questionnaire. Questionnaire for parents and carers. (DOCX 21 kb) [file 12875_2019_936_MOESM2_ESM.docx]

## Parents questionnaire

1. Most cough, cold and flu illnesses are caused by:

❑ Bacteria ❑ Virus

1. Antibiotics are needed for:

❑ Bronchitis

❑ Runny nose with green mucus

❑ Runny nose with yellow mucus

❑ Cough, cold and flu symptoms

❑ Middle ear infection

1. Antibiotics are needed for:

❑ Bacterial infections ❑ Viral infections

Please tick only one box for each statement:

|  | Statements | Strongly disagree | Disagree | Neither agree or disagree | Agree | Strongly agree |
| --- | --- | --- | --- | --- | --- | --- |
|  | I will always want antibiotics for my child’s cough, cold or flu symptoms |  |  |  |  |  |
|  | My child will be sick for a longer period if he/she does not receive an antibiotic for cough, cold or flu symptoms |  |  |  |  |  |
|  | I generally know if my child needs an antibiotic before seeing the doctor for cough, cold or flu symptoms |  |  |  |  |  |
|  | I will go to another doctor if my doctor does not prescribe antibiotics for my child for cough, cold or flu symptoms |  |  |  |  |  |
|  | I usually go to the doctors if my child has been unwell with cough, cold or flu symptoms for longer than 3 days |  |  |  |  |  |
|  | I am more satisfied with the doctor visit if I am prescribed antibiotics for my child with cough, cold or flu symptoms |  |  |  |  |  |
|  | I am always guided by what my doctor recommends for my child with cough, cold or flu symptoms |  |  |  |  |  |
|  | I always have to initiate the discussion of antibiotics before my doctor would be willing to prescribe antibiotics for my sick child with cough, cold or flu symptoms |  |  |  |  |  |
|  | I usually know what I want out of the doctor’s appointment before I go |  |  |  |  |  |
|  | I will take my child to see a doctor if he/she has a high temperature (over 40^O^C) |  |  |  |  |  |
|  | I will take my child to see a doctor if he/she has a temperature (37 ^O^C - 40^O^C) |  |  |  |  |  |

Please tick only one box for each answer:

| I get my advice from: | Never | Rarely | Sometimes | Very Often | Always |
| --- | --- | --- | --- | --- | --- |
| My doctor |  |  |  |  |  |
| Previous experience |  |  |  |  |  |
| Next door neighbour |  |  |  |  |  |
| My mother/relative |  |  |  |  |  |
| Internet |  |  |  |  |  |
